# Real-World Experience with Eptacog Beta for On-Label and Off-Label Indications: The Spanish Experience

**DOI:** 10.3390/ph18111640

**Published:** 2025-10-30

**Authors:** Jose Manuel Martin de Bustamante, María Isabel Rivas-Pollmar, Natalia Acedo, Nora Butta, Victor Jimenez-Yuste, Maria Teresa Álvarez-Román

**Affiliations:** 1 Department of Haematology, Hospital Universitario la Paz, 28046 Madrid, Spain; 2 Department of Haematology, Hospital Universitario la Princesa, 28006 Madrid, Spain

**Keywords:** Eptacog beta, rFVIIa, haemophilia, FVII deficiency

## Abstract

**Background:** Eptacog beta is a novel recombinant activated factor VII (rFVIIa). Preclinical studies have shown the product has a similar profile to eptacog alfa. The PERSEPT 1 and PERSEPT 3 trials proved the efficacy and safety of eptacog beta for surgical prophylaxis and treatment of bleed in patients with haemophilia A or B with inhibitors. This led to approval of the product by the Food and Drug Administration (FDA) and the European Medicines Agency (EMA). Recently, real-world data have been published on the use of eptacog beta on this population which has provided positive outcomes. Given the similar in vitro and in vivo profile of eptacog beta and eptacog alfa positive outcomes could be expected not only in patients with haemophilia and inhibitors, but also in other situations where eptacog alfa is a therapeutic option. **Methods:** We report on the use of this product for on-label and off-label populations in two Spanish hospitals. **Results:** We describe four cases in an on-label population (haemophilia A with inhibitors) and four cases in another population (one with acquired haemophilia and three with mild factor VII deficiency). **Conclusions:** Our experience provides further evidence of the efficacy and safety of eptacog beta for surgical prophylaxis and treatment of bleeding in patients with haemophilia A with inhibitors, but also in those with factor VII deficiency. To our knowledge, this is the first report to describe the use of eptacog beta for factor VII deficiency and acquired haemophilia.

## 1. Introduction

Congenital haemophilia A and B are X-linked coagulation disorders caused by a deficiency in factor VIII (FVIII) and factor IX (FIX), respectively [1]. These conditions are associated with recurrent haemorrhages, most frequently affecting joints, leading to progressive disability if not adequately controlled. The cornerstone of their management is intravenous replacement of the missing clotting factor, either on demand or prophylactically. A major complication with this strategy is the development of neutralising antibodies, or inhibitors, against FVIII or FIX, which render replacement therapy ineffective and complicate both acute and long-term management [1]. Inhibitors are classified as low-titre (LTI) if <5 Bethesda Units (BU) or high-titre (HTI) if ≥5 BU. In patients with HTI, factor replacement is usually ineffective, while in haemophilia B, inhibitor development can also be associated with severe allergic reactions and nephrotic syndrome, further limiting treatment options [2]. For several decades, bypassing agents were the only therapeutic alternative for patients with inhibitors. These included activated prothrombin complex concentrate (aPCC) and eptacog alfa, a recombinant activated factor VII (rFVIIa). Both agents can achieve haemostasis in many situations but involve notable drawbacks. Their efficacy is variable; they have short half-lives requiring frequent intravenous administration; and their use is associated with an increased risk of thrombosis. Furthermore, they are suboptimal for prophylactic use, leaving patients with high bleeding rates and ongoing joint damage.

In recent years, the therapeutic landscape has changed substantially with the development of non-replacement therapies. Emicizumab, a bispecific antibody that mimics FVIII cofactor activity by bridging FIXa and activated factor X (FXa), has revolutionised prophylaxis in haemophilia A with inhibitors. It was approved in 2017 by the Food and Drug Administration (FDA) and in 2018 by the European Medicines Agency (EMA). Additional agents such as concizumab, an anti–tissue factor pathway inhibitor antibody, and fitusiran, an siRNA that reduces antithrombin levels, have also demonstrated efficacy, and received regulatory approval by the FDA for prophylaxis in haemophilia A and B with inhibitors. These treatments significantly reduce bleeding frequency when used as prophylactic treatment, but they cannot be used for the treatment of acute haemorrhage. In such cases, patients with LTI may sometimes respond to higher doses of factor replacement, but those with HTI or refractory LTI require bypassing agents. This situation becomes particularly challenging in patients on emicizumab prophylaxis, as concomitant use of bypassing agents, especially aPCC, has been associated with an increased risk of thrombotic events.

**Figure 1:**
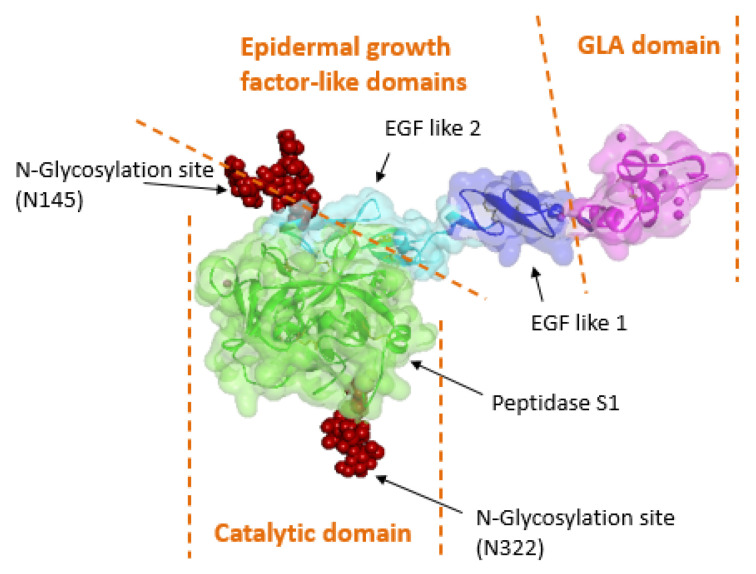
Structure of eptacog beta (published with permission from LFB BIOMEDICAMENTS).

Eptacog beta is a new rFVIIa that shares the same amino acid sequence as endogenous FVIIa and eptacog alfa [3]. This rFVIIa consists of a light and heavy chain, with a total of four domains, as shown in Figure 1. The light chain has three domains: a γ-carboxiglutamic acid (GIa) domain that mediates phospholipid membrane binding, and two epidermal growth factor-like domains that facilitate interactions between rFVIIa and tissue factor (TF), FX, and FIX [3]. The heavy chain contains a C-terminal serine protease domain responsible for enzymatic activity [3]. The structural differences between eptacog alfa and eptacog beta result from their different production, leading to distinct post-translational modifications [3,4].

Functional studies have demonstrated that both molecules act through three principal mechanisms: a tissue factor-dependent pathway activating FIX and FX on the platelet surface, a tissue factor-independent pathway based on binding to activated platelets and generating FIXa and FXa, and an endothelial protein C receptor (EPCR)-dependent pathway that reduces anticoagulant activity [3,4]. The first mechanism is similar for eptacog alfa and eptacog beta [3,4]. For the second mechanism, in vitro studies have shown that eptacog beta has 40% higher maximum binding to platelets at saturation in comparison with eptacog alfa [5].

The last mechanism is based on the binding of the GIa domain to the EPCR, which blocks the activation of protein C and thereby decreases its anticoagulation function [3,4]. Eptacog alfa and eptacog beta display similar affinity to soluble EPCR, but eptacog beta demonstrated a 25 to 40% increased binding activity in a human umbilical vein endothelial cell-based assay [3,5]. Although these in vitro findings suggest a slightly better profile for eptacog beta, the clinical relevance of these differences remains to be established [4].

The clinical efficacy and safety of eptacog beta have been established in phase III trials. The PERSEPT 1 study assessed its use in the treatment of bleeding episodes, while PERSEPT 3 evaluated surgical prophylaxis in patients with haemophilia A or B and inhibitors [6,7]. Both trials showed favourable outcomes, leading to regulatory approval of eptacog beta for the treatment of bleeding episodes and for perioperative haemostatic management in adults and adolescents (≥12 years) with HTI or LTI refractory to factor replacement. Despite these encouraging results, a major limitation is that patients receiving emicizumab prophylaxis were not included, leaving a gap in the available evidence for this increasingly frequent clinical scenario.

More recently, a case series by Youkhana et al. described the successful use of eptacog beta for bleeding management and perioperative haemostasis in patients on emicizumab prophylaxis, supporting its safety in this setting [8]. Given their similarity, eptacog beta could also be expected to provide clinical benefit in other conditions where eptacog alfa has established indications, such as congenital factor VII (FVII) deficiency or acquired haemophilia.

Here, we present the clinical experience of two Spanish centres in the use of eptacog beta in eight patients with congenital haemophilia A or B and inhibitors. Our series includes both on-label and off-label indications and provides real-world data, with particular emphasis on cases in patients receiving emicizumab prophylaxis.

## 2. Results

Table 1 summarises the most important characteristics of all the cases included. Eight patients were treated, four for on-label indications and four for off-label indications. A brief summary of each case is presented below.

### 2.1. On-Label Cases

Patient 1:

A 51-year-old man with mild haemophilia A, a history of LTI, and receiving on-demand treatment by personal choice, was admitted for elective resection of a giant neurofibroma on his right thigh. A single dose of FVIII (50 IU/kg) was administered preoperatively, but provided a suboptimal response (FVIII activity: 3% pre-dose, 36% post-dose). Consequently, the treatment was switched to eptacog beta at 200 µg/kg immediately before surgery, followed by 75 µg/kg two hours later. No intraoperative bleeding was reported, thus permitting a decrease in the rFVIIa dosing interval to every three hours. At 24 h post-procedure, haemostasis was satisfactory, and the dosing interval was further extended to every eight hours. After 48 h, the patient maintained an adequate haemostatic status. Once the bleeding risk was considered low, a higher dose of FVIII (70 IU/kg) was evaluated for maintenance, showing an adequate response. The patient was discharged after adjustment of FVII dose and frequency, and rFVIIa was reserved for emergency treatment. He did not experience any bleeding or thrombotic episodes during hospitalisation or follow-up.

Patient 2:

A 67-year-old man with severe haemophilia A and LTI, on prophylaxis with emicizumab every two weeks, was admitted for elective inguinal hernia repair. A dose of eptacog beta of 75 µg/kg was administered before surgery, achieving haemostasis during surgery, followed by further 75 µg/kg doses every three hours. Twelve hours after surgery, the patient developed hypotension and abdominal pain. An abdominal CT-scan revealed haemoperitoneum with an active bleed through the epigastric artery. The on-call interventional radiologist performed arterial embolisation, successfully controlling the haemorrhage. Initially, eptacog beta was continued at 75 µg/kg every two hours, but administration of FVIII (50 IU/kg) was tested and produced an adequate response (from 0% to 200% FVIII activity despite an inhibitor of 0.4 BU). Due to these results, eptacog beta was replaced with FVIII concentrates at a dose of 50 IU/kg every eight hours, and provided effective haemostasis. The dosing was adjusted according to FVIII activity, and the patient also continued his emicizumab prophylaxis. On the 10th day of treatment with FVIII, the inhibitor titre increased, and the response to FVIII concentrates was lost. This led to the discontinuation of the FVIII, but he remained on emicizumab and rFVIIa as emergency treatment. He was transferred to his referral hospital to continue his post-surgical care, with no thrombotic events reported.

Patient 3:

A 15-year-old boy with mild haemophilia A and a history of eradicated HTI, receiving on-demand treatment, was admitted to the emergency department as he was experiencing severe pain, paraesthesia and complete functional impairment of the left leg. Ultrasound imaging revealed an intramuscular haematoma in the left iliopsoas. Treatment with FVIII concentrates was started on arrival, with a suboptimal response (from 7% of activity to 26%). The patient had been on daily treatment with FVIII concentrates a month before, due to another intramuscular haemorrhage. Given the probable reappearance of the inhibitor, the treatment was switched to eptacog beta at 75 µg/kg every three hours and emicizumab prophylaxis was initiated. Forty-eight hours later, the haematoma was stable, but the pain had only mildly improved, so percutaneous drainage was performed. Eptacog beta at 75 µg/kg was administered before the procedure. After drain placement, rFVIIa frequency was reduced to every six hours for 72 h, every eight hours for seven days, and then every twelve hours for seven days. The patient continued to improve and was discharged after 18 days. He continued to receive emicizumab weekly and eptacog beta 75 µg/kg once daily for a week, then eptacog beta was reduced to 75 µg/kg on the days the patient had motor rehabilitation (three times weekly). After a month, rFVIIa treatment was discontinued, with no recurrent bleeding or thrombotic complications.

Patient 4:

A 64-year-old man with mild haemophilia (baseline FVIII activity of 6%), no history of inhibitor, and receiving on-demand treatment, was admitted due to an intracerebral haemorrhage secondary to a hypertensive crisis. Continuous infusion of FVIII (50 IU/kg/12 h) was initiated, with a good initial response. After one week, the response started to weaken, and HTI was detected. FVIII dose was increased to 100 IU/kg/12 h, with no effect, so treatment was switched to weekly emicizumab and eptacog beta 75 µg/kg every eight hours. After 24 h, the patient experienced decreased level of consciousness and fever and was diagnosed with septic shock after imaging tests ruled out further bleeding. He was admitted to the intensive care unit (ICU), where a jugular central venous catheter was inserted. After 48 h, the patient developed a large haematoma around the catheter, so the frequency of eptacog beta was increased to every four hours. Due to a shortage of eptacog beta after 48 h, the patient’s haemostatic treatment was switched to eptacog alfa at higher doses (100 µg/kg every four hours). Two days later, a re-evaluation CT scan of the brain and neck showed a reduction in both haemorrhages, but also images of cerebral infarctions that had not been present in earlier scans. Due to these findings, rFVIIa was gradually reduced and then discontinued four days after the scan. The patient continued emicizumab and was discharged to a rehabilitation facility one month after admission.

### 2.2. Other Cases

Patient 5:

An 89-year-old man was admitted following a diagnosis of acquired haemophilia at another centre after developing a large intramuscular haematoma on his right arm following cardiac catheterisation. On admission, the patient had 0% FVIII activity with a very high inhibition titre. Clinically, the arm was swollen, severely painful, with haemorrhagic blisters, and complete loss of function. Due to his comorbidities, immunosuppressive therapy with steroids alone (prednisone 1 mg/kg/day) was started, alongside haemostatic treatment with emicizumab and eptacog beta (225 µg/kg initial dose, then 75 µg/kg every three hours). The plastic surgery team was consulted and diagnosed a compartment syndrome with necrotic tissue, indicating surgery, but the patient was not in a suitable condition to undergo the procedure. As an attempt to improve his condition, medical treatment for acquired haemophilia continued. After two weeks with immunosuppression, emicizumab and eptacog beta, no clinical improvement occurred, FVIII activity remained 0% and the inhibitor titre was unchanged. The patient remained unfit for surgery, and, given the lack of therapeutic options, medical treatment was withdrawn, and he was admitted to a hospice.

Patient 6:

A 28-year-old man with mild FVII deficiency (baseline FVII activity 20%) was admitted for elective surgery on a perianal abscess. He received a single dose of eptacog beta at 15 µg/kg prior to surgery, with no bleeding complications. He was discharged two days later, and did not require further treatment or experience bleeding or thrombotic events during follow-up.

Patient 7:

A 45-year-old man with mild FVII deficiency (baseline FVII activity 30%) was admitted for scheduled femur fixation under spinal anaesthesia. A single preoperative dose of eptacog beta at 15 µg/kg was administered, with no bleeding complications. He was discharged three days later, and did not require further treatment or experience bleeding or thrombotic events during follow-up.

Patient 8:

A 69-year-old man with severe FVII deficiency (baseline FVII activity 6%) was admitted to the ICU with septic shock secondary to native aortic valve endocarditis. He required intubation, antibiotics, and an aortic valve replacement. As he developed disseminated intravascular coagulation (DIC), he received fibrinogen (due to Clauss fibrinogen was <100 mg/dL), fresh frozen plasma, and eptacog beta 20 µg/kg prior to surgery. He stabilised, but gradually developed worsening pleural effusion that required drainage, preceded by a dose of eptacog beta 15 µg/kg. As mechanical ventilation continued, a tracheostomy was performed 12 days later, with a preprocedural dose of eptacog beta 20 µg/kg. Over the following weeks we required a central venous catheter insertion, a replacement of the central catheter and a new drainage of the pleural effusion. All the interventions were preceded by a single dose of eptacog beta (20 µg/kg), with no bleeding complications. Unfortunately, while admitted to the ICU, he had an arterial embolism in his left leg, which then became infected. He did not respond to treatment, developed an extensive gangrene and died after two months in hospital.

## 3. Discussion

Although the efficacy of eptacog beta has been demonstrated in several clinical trials, evidence in real-world settings remains limited. A recent case series report was published [8] that includes patients with haemophilia A, showing the safety and efficacy of this rFVIIa in patients receiving emicizumab for bleeding episodes and perioperative management.

In this report, we include the experience from two Spanish centres with eight patients who received eptacog beta for bleeding episodes and perioperative management. Of the eight patients, four were treated according to the indications approved by the FDA and EMA, while four others were treated off- label (one case of acquired haemophilia and three patients with FVII deficiency).

Given the heterogeneity and the small number of patients, these are preliminary data that should be interpreted with caution, as it is not possible to draw solid conclusions from our experience.

### 3.1. On-Label Population

All four patients treated on-label had haemophilia A, and one was on prophylaxis with emicizumab prior to the surgery. Patient 1 and patient 2 were treated with eptacog beta for perioperative management. In both cases, intraprocedural haemostasis was achieved; however, in the second case, haemostatic therapy was changed due to a surgical complication and evidence of an adequate response to FVIII concentrates.

The remaining two patients had mild haemophilia A, received intensive treatment with FVIII concentrates for a bleeding episode. One had developed an inhibitor in the past and underwent successful inhibitor tolerance induction, but later suffered a recurrence. The second patient had no history of inhibitors. Even though the patients had severe haemorrhages, both cases were treated with repeated doses of eptacog beta at 75 µg/kg, as they were also receiving emicizumab and thrombotic risk was a concern. Patient 3 had an excellent response with resolution of the haemorrhagic symptoms, while patient 4 required a switch to eptacog alfa due to a stock shortage of eptacog beta.

In our experience, treatment with eptacog beta in the on-label population was effective, consistent with the literature [4,8,9], without significant bleeding complications during treatment. Although we switched from eptacog beta to other haemostatic treatments in three of the four patients, this was not due to a lack of efficacy in any of the cases. In the eptacog beta clinical trials, it has been suggested that higher eptacog beta doses (225 µg/kg) increase the rate of thrombin generation, promoting the formation of a stable haemostatic clot and resulting in greater efficacy than with lower doses [3]. Of the two initial dose regimens approved for eptacog beta, a starting dose of 75 µg/kg was selected in three of the four cases as they had concomitant emicizumab therapy, and they achieved good haemostatic efficacy.

From a safety perspective, our experience was also favourable, as no thrombotic complications were observed, consistent with published data [4,8,9], although patient 4 experienced a thrombotic event after switching treatment to eptacog alfa.

### 3.2. Off-Label Population

Of the four cases treated off-label, one patient with acquired haemophilia who received eptacog beta 225 µg/kg in combination with emicizumab for haemostatic control. A high-dose regimen of eptacog beta was chosen, as patient 5 had a severe haemorrhagic phenotype with an uncontrolled, life-threatening bleed. Despite the intensive treatment, bleeding could not be controlled, due to the refractoriness of the acquired haemophilia to steroid therapy and the patient’s poor condition, which precluded more intensive immunosuppression.

The other three patients had an FVII deficiency. Patients 6 and 7 received a single 15 µg/kg dose of eptacog beta before surgery, without bleeding complications. This low-dose regimens were chosen as both patients had a mild deficiency and mild bleeding phenotypes. Patient 8 had lower levels of FVII (6%) and required repeated doses of rFVIIa at doses of 15–20 µg /kg prior to different procedures. In this case, a low-dose regimen was chosen because he had a mild bleeding phenotype and multiple thrombotic risk factors. In fact, he had an arterial embolism and, even though we cannot rule out that eptacog beta played a role, we believe that the sum of these high-risk thrombotic factors (severe infection, the long ICU stay, the major heart surgery, etc.) was the most likely cause for the thrombosis.

Eptacog alfa has demonstrated efficacy in the prophylaxis and treatment of bleeding episodes in patients with factor VII deficiency, but no previous evidence published evidence exists for eptacog beta in this population. In our three patients with FVII deficiency treated with low doses of eptacog beta (15–20 µg/kg), the haemostatic effect was sufficient for perioperative management, with no bleeding complications. These favourable results for administering low doses in FVII deficiency could be due the potentially higher affinity of eptacog beta for EPCR compared with eptacog alfa [10]. As mentioned earlier, the thrombotic event observed in one of the patients was probably related to all his associated risk factors, but we cannot rule out a potential contribution of eptacog beta.

Classically, eptacog alfa has been used for haemostatic treatment in patients with acquired haemophilia, but there is no previous published experience on the use of eptacog beta in this context. As previously discussed, even though our patient did not respond as we would have liked, the lack of response to immunosuppression and the severity of the bleeding, which required surgery, made haemostasis difficult to achieve from the outset.

Eptacog beta has been approved for the treatment of haemophilia A and B with inhibitors, but these preliminary data suggest it may also be useful in other conditions where eptacog alfa is approved, such as factor VII deficiency. Treatment of patients with FVII deficiency is still challenging due to the limited number of currently licenced targeted products. The development and analysis of new rFVIIa products, such as eptacog beta, for the treatment of these patients could offer additional therapeutic options and supports the current clinical approach of providing more precise, personalised treatment. In this regard, we are currently conducting an in vitro evaluation of the haemostatic effect of eptacog beta in blood samples from patients with FVII deficiency using various global coagulation assays, and its impact on platelet function in these patients. Stronger studies are needed, as other possible indications of eptacog beta include patients treated with non-replacement therapies who need treatment due to breakthrough bleeds or for patients during the periprocedural period with inadequate response to eptacog alfa, acquired haemophilia and, possibly, invidivuals with thrombopathies.

In addition, based on the results published in the literature and our own experience, eptacog beta is also an attractive option as it provides similar efficacy to eptacog alfa while offering a significant reduction in the costs, as it has been commented in the literature [11,12], reaching a cost reduction of 42% in the experience of Youkhana et al. [8].

## 4. Materials and Methods

This is a retrospective case series from two centres in Spain (la Paz University Hospital and La Princesa University Hospital) involving patients treated between January 2024 and May 2025. The only inclusion criteria were treatment with eptacog beta. No exclusion criteria were established. Haemostatic efficacy for bleeding episodes was defined as effective when bleeding stopped or was significantly reduced within 12 h of initiating treatment, with no additional haemostatic agent required. With regard to surgical procedures, it was considered effective when intraoperative blood loss was within the expected range for a patient without a bleeding disorder, and postoperative bleeding was controlled without unplanned additional therapy or the need for blood transfusions beyond the usual requirements for this type of surgery.

This study was approved by the ethics committee of la Paz University Hospital approval code PI-6242. Patients did not sign informed consent forms as this was a retrospective study where data were collected from medical record. Our ethics committee does not require informed consent to be signed for this type of study.

## 5. Conclusions

Our experience adds to the previous reports that eptacog beta appears to be an attractive therapeutic option for haemophilia A and B with inhibitors, as it has proved to be safe and effective, and even capable of significantly reduce the cost of treatment in certain settings.

Nevertheless, real-world experience is still scarce, and there is a need for more data to confirm the haemostatic and safety results observed in our practice.

Given that patients with inhibitors do not always respond to eptacog alfa, it is important for other therapeutic alternatives to be available, particularly for those patients receiving prophylactic treatment with non-replacement therapies or aPCC, who may be at higher risk of thrombosis.

Real-world data are highly useful when a new product reaches the market. While our data are heterogeneous, we believe they add value to previous publications, as we present the first cases of FVII deficiency and acquired haemophilia treated with eptacog beta. In our experience, eptacog beta may be a viable alternative for FVII deficiency, but these preliminary results must be interpreted with caution until more robust data are available, preferably from randomised trials.

## Figures and Tables

**Table 1:** Summary of cases.

On-Label or Off-Label	Patient	Age	Gender	Disease	Base Factor Levels (IU/dL)	Inhibitor Titre (UB)	Prophylaxis	Eptacog Beta First-Dose (µg/kg)	Eptacog Beta Total Doses	Haemostatic Efficacy	Thrombosis
On-label	Patient 1	51	Male	Mild haemophilia A	3	1.7	No	200	9	Excellent	No
On-label	Patient 2	67	Male	Severe haemophilia A	0	0.4	Emicizumab	75	5	Excellent	No
On-label	Patient 3	15	Male	Mild haemophilia A	7	23	Emicizumab	75	70	Good	No
On-label	Patient 4	64	Male	Mild haemophilia A	6	1.6	Emicizumab	75	21	Excellent	No
Off-label	Patient 5	89	Male	Acquired haemophilia	0	>Upper limit of laboratory	Emicizumab	225	53	Suboptimal	No
Off-label	Patient 6	28	Male	Mild factor VII deficiency	20	Noo	No	15	1	Excellent	No
Off-label	Patient 7	45	Male	Mild factor VII deficiency	30	No	No	15	1	Excellent	No
Off-label	Patient 8	69	Male	Mild factor VII deficiency	6	No	No	20	6	Excellent	Yes

Haemostatic efficacy was defined as excellent, good, and suboptimal based on the definitions of the PERSEPT 1 and PERSEPT 3 trials [6,7]. In surgical cases, excellent was defined as intraoperative blood loss within the expected range for a patient without a bleeding disorder, and postoperative blood loss that was similar to or less than that expected in a patient without a bleeding disorder; no blood component transfusion was required. Good was defined as intraoperative blood loss greater (but not more than 50% greater) than expected in a patient without a bleeding disorder; or postoperative bleeding greater than expected, not explained by a surgical or medical issue other than the bleeding disorder. Suboptimal was defined as an intraoperative blood loss more than 50% greater than expected, or uncontrolled intraoperative bleeding, not explained by a surgical or medical issue other than the bleeding disorder; or postoperative bleeding substantially greater than expected for the same reason. In haemorrhage cases, excellent was defined as full relief of pain and cessation of objective signs of the bleeding episode with the planned treatment. Good was defined as marked improvement of symptoms of the bleeding episode but not completely resolved with the planned treatment. Suboptimal was defined as little to no noticeable effect of the treatment on the bleeding episode or worsening of the patient’s condition.

## Data Availability

The original contributions presented in this study are included in the article. Further inquiries can be directed to the corresponding author.
